# BAP1 Mutations and Pleural Mesothelioma: Genetic Insights, Clinical Implications, and Therapeutic Perspectives

**DOI:** 10.3390/cancers17091581

**Published:** 2025-05-06

**Authors:** Susana Cedres, Augusto Valdivia, Ilaria Priano, Pedro Rocha, Patricia Iranzo, Nuria Pardo, Alex Martinez-Marti, Enriqueta Felip

**Affiliations:** 1 Medical Oncology Department, Vall d’Hebron Institute of Oncology (VHIO), Vall d’Hebron Hospital Universitari, Paseo Vall d’Hebron 119, 08035 Barcelona, Spain; augustovaldivia@vhio.net (A.V.); pedrorocha@vhio.net (P.R.);; 2 Thoracic Cancers Translational Genomics Unit, Vall d’Hebron Institute of Oncology (VHIO), 08035 Barcelona, Spain

**Keywords:** malignant pleural mesothelioma, biomarkers, BAP1, immunotherapy

## Abstract

Pleural mesothelioma (PM) is a locally aggressive tumor associated with asbestos exposure. Despite legislative efforts to regulate asbestos use, its incidence continues to rise in some parts of the world. Chemotherapy and immunotherapy have improved survival in PM patients, but overall survival remains poor. Molecular analysis of PM patients has shown that most alterations occur in tumor suppressor genes, with BAP1 being the most frequently affected. Patients with germline BAP1 mutations have been reported to have a better prognosis, but this is not observed in those with somatic mutations. Interest in developing drugs targeting patients with BAP1 loss has led to several phase II studies in recent years. Unfortunately, initial results have not been very promising. In this review, we conclude that, at this time, with the contradictory results from studies and the limited number of patients evaluated, BAP1, the most commonly altered gene in PM, is not yet suitable for use in clinical practice as a prognostic or predictive factor. Future studies are needed to establish the prognostic or predictive value of BAP1.

## Simple Summary

Pleural mesothelioma is a locally aggressive tumor with a survival rate of around two years. Despite efforts in recent years to identify molecular alterations that could be targeted for treatment, no therapeutic target has been identified so far. BAP1 is the most common molecular alteration in PM, and some studies suggest different sensitivity to treatments in patients with BAP1 loss. The aim of this review is to discuss the predictive value of BAP1 and future targeted therapies.

## 1. Introduction

Pleural mesothelioma (PM) is a rare, locally aggressive, and highly lethal tumor arising from mesothelial cell transformation of the pleura. This malignant tumor is strongly associated with persistent asbestos exposure. The global incidence of PM varies depending on geographical location [1]. The incidence is decreasing in developed countries due to considerable efforts that have been made to clear asbestos from the environment in many countries. However, the incidence of PM continues to increase in many parts of the world where the use of asbestos is not regulated. In addition to asbestos exposure, PM can develop in carriers of germline mutations of BAP1 that are strongly associated with an increased risk of developing PM.

The prognosis of PM is poor, with a median survival of 9–18 months [2]. PM can be classified according to histology from best to worst prognosis into three histological subtypes: epithelioid, biphasic, and sarcomatoid. Histology and stage serve as major prognostic factors in PM [3].

Currently, there are three potential first-line systemic treatment options: chemotherapy based on platinum with pemetrexed, dual immunotherapy with nivolumab and ipilimumab, or a combination of chemotherapy and immunotherapy with pembrolizumab and platinum–pemetrexed. For patients who progress following first-line treatment, clinical guidelines recommend retreatment with platinum and pemetrexed or monotherapy with vinorelbine or gemcitabine.

For more than 15 years, the standard of care for PM has been the combination of cisplatin and pemetrexed, which improves survival from 9.3 months with cisplatin alone to 12.1 months with the combination of pemetrexed [4]. Cisplatin can be replaced by carboplatin in elderly or cisplatin-unfit patients with comorbidities [5,6]. The addition of bevacizumab to chemotherapy showed a benefit in progression-free survival (PFS) and median survival; however, bevacizumab has not received regulatory approval [7].

The introduction of immunotherapy in the first line has been one of the greatest advances in the treatment of PM. The CheckMate 743 study demonstrated that the combination of nivolumab and ipilimumab produced a four-month improvement in overall survival compared to patients receiving standard platinum plus pemetrexed [8]. The 3-year survival rates were 23.2% in the nivolumab plus ipilimumab arm and 15.4% in the chemotherapy arm. However, not all patients responded to immunotherapy. The CheckMate 743 study revealed that patients with non-epithelioid tumors presented a greater immunotherapy effect. In the exploratory biomarker analysis, the median survival was longer for patients treated with immunotherapy with an inflammatory gene signature score, regardless of histology [9].

Multiples studies evaluated the combination of chemotherapy and immunotherapy in the first-line setting [10,11,12]. The IND.227 study showed an improvement of survival for patients treated with the combination of pembrolizumab and chemotherapy, leading to approval by regulatory agencies [10]. The addition of durvalumab to chemotherapy demonstrated favorable results in terms of PFS in a phase II trial, and the results of the randomized phase III study are pending [11]. Finally, the addition of atezolizumab and bevacizumab to chemotherapy in the BEAT-meso trial resulted in an increase in PFS that does not translate into an improvement in median survival [12].

Targeted therapies have been a major advance in the treatment of some tumors, such as lung cancer. In PM, in recent years, there has been a relevant improvement in the understanding of the molecular mechanism underlying tumor biology. Several pathways have been identified, including those related to growth factors, angiogenesis, cell cycle regulation, epigenetic modifications, DNA damage repair, and apoptosis [13]. However, despite promising preclinical results, most clinical trials evaluating potential targeted drugs have yielded unsatisfactory results.

The most frequently mutated genes in PM are tumor suppressor genes, with mutations in BAP1, NF2, CDKN2A, TP53, and SETD2 being the most commonly altered genes [14,15,16,17]. BRCA1-associated protein-1 (BAP1) is a tumor suppressor gene encoding a deubiquitinase with roles in several cellular processes involved in oncogenesis (cell cycle regulation, cellular differentiation, cell death, and DNA damage response) [18]. In PM, BAP1 as tumor suppressor contributes to mesothelioma development, and loss of BAP1 leads to uncontrolled cell growth. Moreover, BAP1 controls cell cycle checkpoints, enhancing mesothelioma progression when BAP1 is lost. Finally, BAP1 in PM plays a role in the DNA damage response, and loss of BAP1 leads to accumulation of DNA damage that may be sensitive to PARP inhibitors. As previously noted, PM may originate from either somatic or germline events, with BAP1 alterations reported in both settings. Currently, it remains unclear whether the oncogenic mechanisms driven by BAP1 mutations differ between tumors arising from hereditary predisposition and those induced by environmental exposures.

Although the reported frequency for somatic BAP1 loss of function varies, studies show BAP1 is the most commonly altered gene in PM, and BAP1 is inactivated in approximately 60% of PM and up to 80% in the case of epithelioid cancers [19,20,21,22].

However, the critical role of BAP1 as a tumor suppressor in PM remains incompletely understood, and BAP1 is not fully implemented as a clinical prognostic or predictive biomarker yet. Understanding BAP1 status in PM can provide insight into prognosis and potential therapeutic strategies. This review aims to explore whether BAP1 can serve as a reliable prognostic biomarker and a potential therapeutic target in PM.

## 2. BAP 1 Gene

The BAP1 gene was identified in 1998 as a powerful tumor suppressor gene, and, in 2011, its association with germline and somatic carcinogenesis of PM was defined. Human BAP1 is a 729 amino acid predominantly nuclear deubiquitinase encoded on chromosome 3p21.1 [23]. The gene is expressed in all normal mesothelial cells [24]. It forms multiprotein complexes that regulate key cellular pathways, including genomic stability, DNA repairs, and cell death [18,21].

BAP1 inactivation usually arises from somatic single nucleotide variants but may also arise from chromosomal rearrangement, gene fusion, and splice-site alterations [25]. The inactivation of BAP1 alters chromatin remodeling and DNA repair and regulates cell metabolism. BAP 1 mutations appear to be an early event in mesothelioma pathogenesis, representing the most common molecular alteration in reported cases of mesothelioma in situ [26,27].

**Figure 1:**
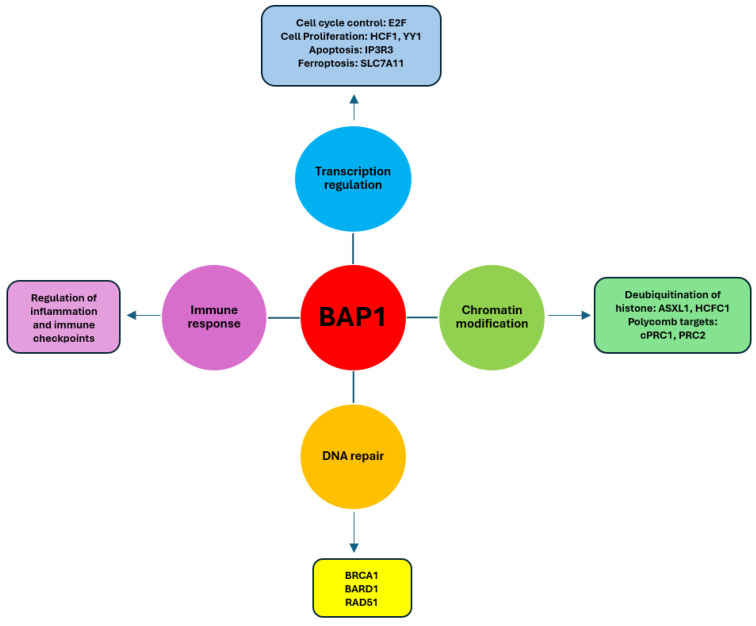
Illustration of the cellular processes regulated by BAP1 in PM. BAP1 is involved in the regulation of transcription, chromatin modification, DNA repair, and immune response. Alterations in BAP1 can disrupt any of these processes. Intervening in the target molecules of these processes could control BAP1-dependent tumors.

BAP1 alterations have a profound effect on PM biology, interacting with multiple genes and epigenetic mechanisms to develop the tumor (Figure 1) [21,27]. BAP1 regulates the homologous recombination system, thus causing genomic instability when BAP1 loss occurs. BAP1 is involved in epigenetic deregulation, and the loss of BAP1 in mesothelioma alters histone modification patterns, altering gene expression. Finally, at the metabolic level, BAP1 is involved in mitochondrial function and metabolism, and the loss of BAP1 promotes PM growth. BAP1 alterations can co-occur with other alterations such as co-mutations with NF-2, which lead to a loss of Hippo pathway signaling and increased proliferation; co-mutations with TP53, which lead to more aggressive tumors; and co-mutations with SETD2, which affect chromatin remodeling. An evolutionary analysis suggests that BAP1 loss occurs early in the evolution of PM, whereas NF2 loss occurs later in the disease progression [28].

BAP1 loss is seen in up to 60% of PM, and the majority of BAP1 mutations in PM are somatic (70–80%), whereas germline BAP1 mutations are less common and associated with less aggressive tumors than those in PM harboring somatic BAP1 mutations [29]. BAP1 alterations are found more often in epithelioid (50–70%) than in sarcomatoid (20%) tumors [15,30].

BAP1 mutations may influence response to therapy. BAP1-deficient tumors have defective DNA repair, making them potentially more sensitive to DNA-damaging agents like cisplatin. Moreover, BAP1 mutations are associated with an immunogenic tumor microenvironment and with alteration of tumor antigen presentation, making tumors with BAP1 loss good candidates for immunotherapy.

The standard and most widely used method to assess BAP1 is immunohistochemistry (IHC). BAP1 serves as valuable biomarker in PM diagnosis, helping distinguish it from reactive mesothelial proliferations and in the differential diagnosis with certain carcinomas [31,32]. Furthermore, BAP1 loss is not specific for PM, and it may be seen in clear cell renal carcinoma, thymic carcinoma, and melanoma [20,22,33,34,35,36,37]. Normal cells show nuclear BAP1 staining, while tumor cells with BAP1 loss show complete absence of staining. BAP1 loss is very homogeneous in neoplastic PM cells in most cases [38]. Four BAP1 staining patterns have been described: single nuclear positivity, single cytoplasmic staining positivity, single absent staining, and combinations of these staining patterns [38].

BAP1 mutations can also be detected using an integrated genomic approach detecting somatic and germline mutations [21]. However, it is not always available in routine clinical settings. The absence of BAP1 correlates with BAP1 inactivating mutations, since almost 100% of inactivating mutations cause the loss of the nuclear localization signal located at the carboxy-terminus of the BAP1 protein [21]. However, in a study, BAP1 loss IHC analysis showed that 25% of PM with negative nuclear IHC staining of BAP1 were negative for BAP1 gene mutations [20,39,40]. This discrepancy appeared related to methodological approaches used to detect genetic alterations.

## 3. BAP1 Germline Mutations

Several studies have identified germline BAP1 mutations in families with a high incidence of mesothelioma, suggesting a hereditary predisposition to the disease [41,42,43]. Based on the observation that several cases of PM occur in some families, a genetic study of two human families whose members showed a high incidence of PM identified germline BAP1 mutation as responsible for the familial aggregation of PM [29]. This study showed for the first time that BAP1 mutations are associated with a novel hereditary cancer syndrome that predisposes to mesothelioma, uveal melanoma, and clear cell renal cell carcinoma, among other cancers (Table 1) [44].

Pathogenic germline BAP1 mutations are inherited in an autosomal dominant pattern with a 50% chance to inherit the mutation family members [45]. BAP1 germline mutations are observed in 9–12% of PM patients, although more commonly detected in younger-age patients and in patients with tumors related to BAP1 tumor predisposition syndrome [46]. It has been described that 30% of carriers of germline BAP1 mutations have developed mesothelioma, underscoring the key role of BAP1 preventing the malignant transformation of mesothelial cells [21,44]. Considering the high somatic rate of BAP1 alterations, germline testing should be undertaken when the variant allele frequency is higher than 10% [47]. Identification of BAP1 mutations in individuals with a family history of PM can facilitate early screening and surveillance, allowing for earlier detection and intervention. BAP1 families require genetic and oncological counseling to handle cancer risk management and undergo routine testing for at-risk family members. The recommendations for carriers of pathogenic germline BAP1 mutations include periodic dermatologic and ophthalmologic examination and chest magnetic resonance imaging (MRI) [44].

Regarding the prognosis, germline BAP1 mutations predispose carriers to developing mesothelioma; however, these same mutations render mesotheliomas less aggressive and possibly more sensitive to chemotherapy [48]. Median survival in patients with BAP1 germline alteration is around 5–7 years, with 26% of patients surviving 10 or more years [44].

## 4. Prognostic Value of BAP1 in PM

The prognostic value of BAP1 has been analyzed in several studies, with controversial results (Table 2). A retrospective review of 229 mesothelioma patients aimed to determine the clinicopathological significance of BAP1 IHC in mesothelioma [49]. The cohort included 46% of patients with negative staining for BAP1, and an improvement on median survival of 16 months versus 6 months was detected in BAP1-loss patients. In another series of 117 patients, BAP1 expression was associated with a longer survival when BAP1 expression is lost [19]. In a study including 55 only epithelioid tumors, BAP1 loss was detected in 60% of cases and significantly associated with longer survival (14 months versus 11 months for BAP1 retained) but not with longer PFS [50]. The association of BAP1 loss with improved survival was found in the univariate analysis but not confirmed in the multivariate analysis. The RAMES trial, a phase II study, assessed the efficacy and safety of ramucirumab (anti-VEGFR) combined with gemcitabine in patients with pretreated PM [51]. The study was positive, showing an improved survival after first-line chemotherapy. The trial included a study of correlations between genetic alterations and outcomes in 164 patients, and BAP1 variants were associated with longer PFS after platinum pemetrexed, with a possible prognostic role at the first line of treatment. The mechanism by which the loss of BAP1 influences the prognosis is not well understood. A preclinical study discovered that, when BAP1 is mutated, the levels of HIF-1α are significantly reduced and suggested that the improved prognosis observed in PM with BAP1 mutations may be linked to the reduced HIF-1α levels in the tumor cell and in the microenvironment [52].

The previously reviewed studies have suggested that BAP1 is a favorable prognostic factor for survival; however, two additional studies have reported opposite results, and a meta-analysis was unable to confirm the prognostic value of BAP1. In a cohort of 81 patients of matched cytology and surgical specimens, BAP1 loss was significantly associated with poor survival [53]. Finally, a single-center analysis including a systematic review and meta-analysis confirmed that BAP1 expression is not an independent prognostic factor for PM patients and should not be considered without considering tumor histology. The single-center cohort included 60 PM, with 82% of patients BAP1-negative. The median survival was 14.8 months and 18.1 months for negative and positive BAP1 expression, respectively. The multivariate analysis did not find any differences among the two groups. Similarly, the meta-analysis that included 698 patients showed no differences in terms of survival according to BAP1 status [54].

Different scenery represents patients with germline BAP1 mutations. As previously reviewed, germline BAP1 mutations have been linked with longer survival in PM; in fact, germline BAP1 mutations are the first molecular prognostic factor in PM [55]. The tumors with this mutation present a less aggressive phenotype of PM arising in the context of BAP1 cancer syndrome [55,56]. A comparative analysis for survival using data from SEER identified 23 mesotheliomas with germline BAP1 mutations, including peritoneal and pleural mesothelioma [55]. The median survival was 7-fold improved in patients with germline mutations compared with patients with sporadic tumors. In the largest cohort of patients with germline alterations evaluated, patients with germline mutations were younger at diagnosis and more likely to be female. After adjusting for age and gender, genotype had an independent and significant effect on survival [56].

The causes of the survival differences between these studies are unknown. On the one hand, these may be due to methodological differences in immunohistochemical staining. Second, the antibodies used in these studies were different. Furthermore, differences in the technique used to assess BAP 1 expression by IHC or miRNA could have altered the results. Moreover, some studies used tumor tissue arrays to express the results. Finally, the small number of patients in some studies and possible imbalances in the series regarding prognostic factors such as histology or stage could have altered the results.

## 5. BAP1 as a Predictive Biomarker in PM

It has been suggested that BAP1 alterations may serve as a predictive candidate biomarker for PM to improve disease stratification and therapy (Table 3). Due to its involvement in homologous reparation repair (HRR), BAP1 has been studied as biomarker for response to chemotherapy. For more than two decades, the standard first-line treatment for patients with mesothelioma has been the combination of platinum and pemetrexed. Based on the biological function of BAP1 and longer survival in patients with germline mutations, a large series study investigated whether patients with PM with tumors harboring BAP1 loss as determined by IHC have improved survival compared to a first-line chemotherapy with platinum and pemetrexed [48]. Most of the tumors in this cohort had loss of BAP1 (60%). The survival analysis revealed a significant survival advantage in those patients with BAP1 loss in tumors, with more than 10 months’ increase in survival (20.1 versus 7.3 months) in patients treated with platinum and pemetrexed in the first-line setting. In contrast, patients with retained BAP1 who received chemotherapy had similar median survival to those who received no active treatment. This study represented the first evidence for a predictive biomarker for survival after primary chemotherapy in PM.

A large study with a cohort of 385 with pleural and peritoneal mesothelioma evaluated whether inherited loss of function mutations in patients with mesothelioma impact overall survival following platinum-based chemotherapy [56]. The series included 66% of PM, and genetic profiles revealed 12% of patients harboring damaging mutations, with BAP1 being the most frequently altered. The data of the study suggest that, compared with patients with no germline mutation, survival was significantly better for patients with a mutation in BAP1 or for patients with a mutation in any of the targeted genes.

Therefore, it can be concluded that, based on the results of these two studies, BAP1 could be a predictor of survival in patients treated with platinum. However, these two studies are retrospective analyses, and prospective studies would be necessary to validate the results.

The mechanism by which BAP1 loss provides a survival advantage to chemotherapy is not clear, but it is considered that a hampered DNA repair system is unable to repair platinum-induced DNA damage leading to apoptosis [57].

After first-line treatment, there is no approved chemotherapy treatment. However, based on small phase II studies and case series, gemcitabine and vinorelbine have been recommended as therapies for platinum progression [58,59]. The potential predictive value of BAP1 for chemotherapy treatment recommended in the second or third line has been evaluated in preclinical studies, case series, and retrospective reviews of clinical studies. The chemosensitivity of gemcitabine on human mesothelioma cell lines carrying BAP1 wild-type versus mutant cells was evaluated in a preclinical study [60]. The analysis determined that BAP1 mutant cells were significantly less sensitive than BAP1 wild-type cell lines to gemcitabine, and silencing of BAP1 significantly increased resistance of mesothelioma cells to gemcitabine. Significantly decreased DNA damage in the form of double-strand breaks was observed in gemcitabine-treated BAP1 mutant cells, compared to wild-type BAP1. Similarly, a second preclinical model investigated whether loss of function of BAP1 could be exploited for targeted therapy. They found that gemcitabine was more cytotoxic in BAP1 wild type cell lines compared to BAP1-mutated cell lines, suggesting a potential clinical application where BAP1 status could serve as predictive biomarker for treatment with gemcitabine [61].

In the MS01 evaluating the addition of chemotherapy to active symptom control, survival, or quality of life, although, in the global analysis, the addition of chemotherapy did not improve survival, exploratory analyses suggested that vinorelbine had some benefit in terms of PFS [62]. A retrospective response analysis of BAP1 expression in archival biopsies taken from patients in this study revealed a small and non-significant survival disadvantage associated with BAP1 expression in tumors from patients treated with vinorelbine, suggesting that BAP expression may modify response to vinorelbine in PM [63].

As will be discussed later, the efficacy of PARP inhibitors in PM is not clearly related to BAP1 status. The phase II study with olaparib found a limited efficacy, but, in a subset analysis, the survival was longer for those patients with germline BAP1 mutations compared with the wild type [64]. The MiST1 phase II trial, which evaluated the efficacy of rucaparib in patients with PM harboring BAP1 deficiency, demonstrated that BAP1 status assessed by ICH did not predict response to PARP inhibition [65].

In conclusion, current evidence suggests that germline BAP1 mutations possess predictive value for platinum-based therapies. However, their predictive significance in patients harboring somatic BAP1 mutations remains unclear. With regard to second-line chemotherapy, vinorelbine has been associated with a non-significant improvement in survival among patients exhibiting BAP1 loss, based on retrospective analyses. The predictive role of gemcitabine has thus far only been investigated in preclinical settings. Concerning PARP inhibitors assessed in clinical trials, the data regarding the predictive value of BAP1 remain inconclusive and contradictory.

## 6. Efficacy of Immunotherapy in BAP1-Mutated PM

Immunotherapy has been established as a standard first-line treatment for PM with the approval of the combination of nivolumab plus ipilimumab and chemotherapy plus pembrolizumab [8,10]. In solid tumors, PD-L1 has been proposed as a predictive factor for immunotherapy. In PM, PD-L1 is expressed, at low levels, in 40–45% of patients and mainly in the sarcomatoid subtype, but its predictive role in PM is controversial [66,67,68]. Although early phase I and II studies have found high activity of immunotherapy for PD-L1-positive tumors, the phase III studies did not find differences in PD-L1-expressing patients compared to PD-L1-negative patients [8,10,11]. Regarding other biomarkers predictive of response to immunotherapy, mutations in genes of mismatch repair (MMR) predict efficacy of immunotherapy in solid tumors, but, in PM, MMR alterations are rare [69].

Recent studies have investigated the relationship between BAP1 deficiency and the tumor immune microenvironment in PM. In a genomic analysis including 19 peritoneal mesothelioma, BAP1 loss was associated with lower PD-L1 expression [70]. Similar results were detected in a large study with 1294 samples from pleural and peritoneal mesothelioma analyzed through NGS [71]. In this cohort, BAP1 was altered in 44% of cases and TMB was consistently low in BAP1-altered tumors and wild-type mesotheliomas. The percentage of PD-L1 high tumors was significantly higher in BAP1 wild-type versus BAP1-altered tumors. Additionally, homologous recombination repair alterations were less prevalent in the BAP1-altered tumors compared with the wild type. However, a third preclinical study with murine PM has shown opposite results [72]. In the study, tumors with BAP1 loss exhibited enriched immune-associated pathways (interferon alpha/gamma, dendritic cells, immune checkpoint receptors, and T-cell inflammation) and were associated with a better immunotherapy outcome. The findings suggest that BAP1-deficient PM may have enhanced sensitivity to immunotherapy.

A clinical series of BAP1-altered patients evaluated the outcomes of systemic therapies in PM [73]. In total, 45 patients were treated with chemotherapy, immunotherapy, or PARP inhibitors. No responses were seen with PARP inhibitors and no differences in response rates or PFS with chemotherapy or immunotherapy according to BAP1 status, concluding that patients with BAP1-altered tumors presented a similar efficacy of chemotherapy and immunotherapy.

The relations between BAP1 loss and PD-L1 expression in mesothelioma was explored in clinical trials. PrE0505 study is a phase II study for previously untreated PM [11]. Patients received durvalumab in combination with chemotherapy, and the median survival for all enrolled patients was 20.4 months, longer than the historical controls. A genomic exploratory analysis of the study found that patients with pathogenic germline loss of functions in PM susceptibility genes had significantly prolonged survival. Interestingly, patients with sporadic BAP1-mutant PM harboring heterozygous somatic inactivating mutations did not have better response or survival, suggesting that immune surveillance mechanism differ in the context of germline BAP1 deficiency. Moreover, in the study, although patients with BAP1 somatic mutations with epithelioid histology had higher intratumoral CD8 T cell infiltration, this finding did not translate into an increase in survival.

A phase II trial investigating niraparib plus dostarlimab for patients with PD-L1 >1% and HRR mutations demonstrated a lack of benefit in BAP1-mutant patients [74]. The median PFS in the cohort of somatic BAP1-mutant PM was 2.9 months, and the only patient with BAP1 germline mutation experienced stable disease for 14 months. The efficacy of the combination in the trial was lower as compared with that observed with immunotherapy unselected patients, highlighting a potential negative predictive role of somatic BAP1 mutations with the combination of PARP inhibitor and immunotherapy.

Apart from these two phase 2 trials, none of the reported first-line phase III trials (CheckMate 743, IND227 and BEAT-meso) have, so far, presented an analysis on the possible impact of immunotherapy treatment considering BAP1 status.

Therefore, based on the results of these studies with a limited number of patients, we cannot conclude at this time that BAP1 plays a predictive role in response to immunotherapy. Currently, BAP1 status should not be considered when initiating immunotherapy treatment in a patient with PM. Future prospective immunotherapy trials are needed to validate BAP1 deficiencies as a biomarker predictive of response to immunotherapy.

## 7. Emerging Targeted Therapies for BAP1 PM

At this time, there are no targeted therapeutics approved for the treatment of PM. Preclinical data have provided evidence for a host of potential therapeutic targets highlighting EZH2 and PARP as the most promising targets.

### 7.1. EZH2

The enhancer of zester homolog 2 (EZH2) is a histone methyltransferase that catalyzes Polycomb repressive complex 2 (PRC2), leading to chromatin remodeling [75]. EZH2 is required for the physiological differentiation of lung mesothelium, and its dysregulation is associated with carcinogenesis [75]. It has been found to be overexpressed in 85% of PM, more frequently in sarcomatoid tumors [71,76]. The WHO recognized EZH2 as a diagnostic marker allowing to distinguish between PM from benign mesothelial proliferation [77].

EZH2 is upregulated in BAP1-deficient tumors [75]. Several preclinical studies showed increased sensitivity to EZH2 inhibition in BAP1-deficient models compared to wild-type mesothelioma. In a mouse model study, BAP loss resulted in an increase in EZH2 and PCR2, and BAP1 loss cells were sensitive to EZH2 inhibition, providing evidence for the potential role of BAP1 in mediating tumor sensitivity of EZH2 inhibition [75]. Similar results were found in another preclinical model that demonstrated that BAP1 loss induced EZH2 upregulation, and mice with BAP1 loss had shorter survival [78]. A third preclinical analysis in cell lines confirmed that the inhibition of PCR2 induced an antitumor effect, suggesting a targeted therapy for BAP1-deficient PM [65,79].

A phase II open-label, multicenter study with tazemostat, an oral selective inhibitor of EZH2, in a trial with 74 relapsed patients, showed modest activity [80]. In the trial, 99% of patients had BAP1 protein loss assessed using immunohistochemistry, and 38% of patients had missense or indel inactivating BAP1 mutations. The ORR was 3% with a 12-week disease control rate (DCR) of 51%, median PFS was 18 weeks, and median survival was 36 weeks. The modest responses do not seem to be related to BAP1 deficiencies with a 12-week DCR of 54% in the cohort with BAP1 inactivation. Data from this clinical trial suggest that EZH2 inhibition is a potential target for therapeutic intervention in PM; however, the depth and duration of response is suboptimal.

In view of the modest activity of EZH2 inhibitors as single agents, therapeutic combinations have been tested in preclinical studies. In vitro, studies have shown a highly synergistic potential for the combination of EZH2 and FGFR with ATM inhibitors [81,82]. A human and murine preclinical model for mesothelioma and uveal melanoma evaluated the efficacy of the synergistic potential of combining inhibition of EZH2 and FGFR [81]. The efficacy of the inhibition was confirmed in vitro and in mouse models. The data showed sensitivity to dual inhibition in BAP1-deficient tumors but not for BAP1-proficient subtypes. These findings suggest a potential therapeutic strategy combining the inhibition of EZH2 plus FGFR in BAP1-deficient tumors. In another study of a combination, an EZH2 inhibitor was evaluated in combination with a panel of 20 anticancer compounds [82]. A highly synergistic potential for the combination of ATM inhibition with EZH2 inhibition was observed only in BAP1-deficient tumors but not in BAP 1-proficient tumors. Finally, in a spheroid model, the inhibition of EZH2 led to an increase in the expression of chemokines for cytotoxic immune cells, suggesting that EZH2 inhibitors may act synergistically with immunotherapy [83].

In summary, although EZH2 is expressed in most PM patients, and preclinical studies have suggested activity of EZH2 inhibition in patients with BAP1 loss, clinical trials have not confirmed these preclinical data. At present, EZH2-targeted therapies are not in development in PM patients.

### 7.2. PARP

When DNA is damaged by asbestos, the rate of poly ADP ribose polymerase (PARP) increases for DNA repair, and, in theory, PARP inhibitor could have good potential for the treatment of PM, at least for patients with homologous recombination (HR) gene changes [83]. In preclinical studies, PARP inhibitors had a toxic effect on cell lines, although this effect was independent of the BAP1 mutation [84].

**Table 3:** BAP1 predictive biomarker.

Author	Treatment	Number Patients	Predictive Role	Outcome
Louw A (J Thoracic Ongol 2022) [48]	Platinum-pemetrexed	114	+	60% of p BAP1 loss BAP1 loss associated with increased survival (20 vs. 7 m)
Hassan R (PNAS 2019) [56]	Platinum-based	385	+	Pleural and peritoneal mesothelioma Germline BAP1 loss associated with increased survival (7.9 years)
Guazzelli A (Int J Mol Science 2019) [60]	Gemcitabine	Preclinical	+	BAP1 mutant cells less sensitive to gemcitabine
Okonska A (Mol Cancer Therap 2020) [61]	Gemcitabine	Preclinical	+	BAP1 mutant cells less sensitive to gemcitabine
Kumar N (Lung Cancer 2019) [63]	Vinorelbine	89	No significant	BAP1 loss associated with non-significant increases in survival (11 vs. 5 m)
Forde P (Nature Med 2021) [11]	Durvalumab + chemotherapy	55	+	Germline BAP1 alterations associated with longer survival
Zauderer M (Lancet Ongology 2022) [80]	Tazemostat	74	No differences	No differences DCR (54% in BAP1 loss vs. 51% all p)
Ghafoor A (JTO Clin Res Rep) [64]	Olaparib	23	+	Germline BAP1 mutation associated with decreased PFS (3.3 vs. 3.6 m) and survival (8.8 vs. 9.6 m)
Fennell D (Lancet Resp Medic 2021) [65]	Rucaparib	26	+	All p BAP1- or BRCA1-deficient DCR at 12 week 58%
George T (JCO Precis Oncol 2024) [85]	Niraparib	37	+	Solid tumors BAP1 mutant associated with increased PFS (6.7 vs. 1.8 m)

Abbreviation: PFS: progression free survival; DCR: disease control rate.

A few clinical trials have explored the activity of therapeutic effects of PARP inhibitors. With the hypothesis that patients with PM carrying germline or somatic BAP1 mutations or other DNA repair genes deficiencies may clinically benefit from olaparib (PARP inhibitor), a phase II trial investigated the efficacy of olaparib in patients with previously treated PM [64]. The study included 23 patients with pleural or peritoneal mesothelioma, irrespective of BAP1 or BRCA1 status. The primary objective of the study was not met, with only one patient having a partial response, an ORR of 4%, median PFS 3.6 m, and median survival 8.7 months. In the trial, 42% of patients had somatic BAP1 mutations, while 17% of patients had germline BAP1 mutations. The analysis of BAP1 mutation status gives an antithetic result: germline BAP1 mutations were associated with lower PFS and survival compared to somatic ones (2.3 vs. 4.1 and 4.6 vs. 9.6 months). These results suggest that BAP1 status is not a determinant of sensitivity to PARP inhibitors, and patients with germline mutations have worse outcomes.

The MiST1 trial showed modest activity of PARP inhibitors in PM patients with BAP1 and BRCA2 alterations [65]. MiST is an open-label parallel-arm phase 2 trial which explored the efficacy of rucaparib, a PARP inhibitor, in patients with PM with BAP1-deficient or BRCA1-deficient mesothelioma pretreated with chemotherapy. The study included 26 patients, and 38% of patients were BAP1- and BRACA1-negative, 89% of patients were BAP1-negative, and 50% of patients were BRCA1-negative. The DCR at 12 weeks was 58% and at 24 weeks was 23%. Based on a post-hoc analysis, rucaparib showed a median PFS of 17.9 weeks and survival of 41.4 weeks. Rucaparib demonstrated only modest antitumor efficacy in patients with mesothelioma, and losses of BAP1 or BRCA1 were not predictive of response to rucaparib.

A phase II trial evaluated the PARP inhibitor niraparib in patients with solid tumors, including mesothelioma, harboring a BAP1 mutation or other DDR genes [85]. The activity of niraparib was modest, with only one partial response. However, 78% of BAP1-mutant patients showed clinical benefit with treatment. Currently, the NERO trial is evaluating the activity of niraparib in PM in an ongoing phase II study of niraparib versus active symptom control in patients with pretreated mesothelioma.

Therefore, the antitumor activity of PARP inhibitors in PM seems to be limited. There are in vitro observations showing the synergistic effect of anticancer drugs in PM cell lines, for example olaparib and rucaparib plus cisplatin has been effective in the death of PM cell lines [86,87]. BAP1 is linked to an inflamed tumor microenvironment and the infiltration of cytotoxic T cells, suggesting a possible synergistic activity of the combination of immunotherapy and PARP inhibitors [88,89]. The UNITO-001 trial investigated niraparib in combination with dostarlimab (anti-PD-1) for pretreated patients with non-small cell lung cancer or PM [75]. Somatic mutations of BAP1 were detected in 11 PM and germline mutation of the BAP1 gene in one patient. PFS, the primary objective, and survival were 3.1 and 4.2 months, respectively. The median PFS in the cohort of somatic BAP1-mutant PM was 2.9 months, and the patient harboring a germline mutation in the BAP1 gene experienced stable disease with a treatment duration of 14.1 months. Considering the lack of clinical benefit observed in BAP1 mutated patients, the enrollment of this cohort was stopped.

In summary, despite BAP1’s association with the DNA repair system, clinical trials with PARP inhibitors have not confirmed the potential success of these therapies in patients with PM. Only niraparib has demonstrated some activity in patients with BAP1 loss, and a study is currently underway with 35 PM patients with DNA repair system alterations to confirm these results. Future research is aimed at exploring other targets involved in the DNA repair pathway, such as ATR inhibitors.

### 7.3. Other Potential Actionable Targets

Different strategies for inhibiting overexpressed proteins in PM have been analyzed in preclinical studies, with poor results.

The overexpression of EGFR has been described in PM, particularly in epithelioid tumors, but EGFR mutations are rare [14]. EGFR expression is implicated in PM oncogenesis, and treatment of EGFR expressing mesothelioma cell lines with gefitinib caused significant growth inhibition [90,91,92]. However, phase II studies that have evaluated the efficacy of EGFR tyrosine kinase inhibitors erlotinib and gefitinib in patients with PM did not demonstrate clinical activity [93,94]. In a phase II trial with gefitinib in previously untreated pleural or peritoneal mesothelioma, although 97% of patients had EGFR overexpression, gefitinib was not active, with a median survival of 6.8 months [93]. Another phase II trial of erlotinib in 64 patients, previously untreated, evidenced no objective responses and 42% of SD, with a median survival of 10 months. In the analysis of tumor tissue, 65% of tumors had EGFR staining 2+ to 3+, and there was no correlation between EGFR and outcomes [94]. These studies have not evaluated the activity of tyrosine kinase inhibitors in relation to BAP1 expression.

FGFR is overexpressed in some PM, particularly in the sarcomatoid subtype. In a preclinical model, mesothelioma cell lines with BAP1 protein loss were sensitive to inhibition of FGFR [95]. Dovitinib, a multiple tyrosine receptor kinases inhibitor, in a phase II with 12 patients previously treated with chemotherapy, showed minimal activity with several early progression events [96]. Similarly, another phase II with AZD4547, an oral tyrosine multi-kinase FGFR1-3 inhibitor, demonstrated no activity in the second or third line [97]. Despite the limited efficacy results of FGFR and EZH inhibitors in patients with mesothelioma, the efficacy of the combination was studied in preclinical studies. BAP1 loss was associated with enhanced sensitivity to FGFR inhibition, suggesting BAP1 loss as a potential biomarker for FGFR inhibition [95].

BAP1-loss mesothelioma often has low arginine expression, making it more likely to respond to arginine deiminase (ADI-PEG20) [98]. In a mesothelial cells study, BAP1 loss was associated with arginine metabolism, most notable in epithelioid. The loss of BAP1 expression was associated with resistance to ADI-PEG20 [98]. The ATOMIC-Meso, a phase II-III trial evaluated the efficacy of adding ADI-PEG20 or placebo plus chemotherapy in the first line of PM [99]. The study included 249 patients and showed longer survival in the ADI-PEG20 group (9.3 months versus 7.7 months in placebo) and improvement in PFS (6.2 to 7.4 months). In the study, no efficacy results based on BAP1 expression have been reported so far.

## 8. Future Perspectives

Although BAP1 alterations are the most frequent molecular changes in patients with PM, efforts to develop effective therapeutic strategies for these patients have been unsuccessful so far. However, since BAP1 is the most common molecular alteration in these patients, it is essential to continue researching specific therapies that could improve patient outcomes.

Further preclinical research is needed to understand the mechanisms by which BAP1 regulates tumor suppression and the DNA damage response in PM patients. Targeted therapies against BAP1 alterations in PM patients have not been effective to date. Investigating the development of predictive biomarkers for the response to BAP1-targeted therapies could improve patient selection and treatment efficacy. Exploring the interaction of BAP1 with other frequently altered pathways in PM patients, such as the Hippo pathway or TP53 alterations, could enhance the effectiveness of therapies targeting these pathways.

Although there is a connection between BAP1 alterations and tumor immune evasion, BAP1 alterations alone do not appear to be a reliable biomarker for therapy efficacy in PM patients. Research should continue evaluating, at the preclinical level, whether immunotherapy response improves in patients with BAP1 loss and, at the clinical level, through sub-analysis of efficacy data from phase 3 clinical trials in patients with BAP1 alterations.

## 9. Discussion

PM is a locally aggressive disease associated with asbestos exposure. It was expected that, due to policies limiting asbestos use, its incidence would have plateaued by 2020. However, unfortunately, not all countries have been able to adopt these policies, and the incidence continues to rise in some parts of the world. The prognosis of the disease varies, with survival rates generally below two years. Immunotherapy has been established as a new first-line treatment option, improving survival by approximately four months compared to chemotherapy [8,10]. However, not all patients benefit from immunotherapy, highlighting the unmet need for predictive treatment markers.

Over the past decades, both molecular studies and clinical trials exploring new therapies for PM patients have intensified. However, the results remain largely disappointing. Unlike other thoracic tumors, somatic mutations in PM are rare, often undetected by small NGS panels, and currently not targetable with directed therapies [14]. The BAP1 mutation is the most frequently reported genetic alteration in PM patients. This gene is associated with chromatin regulation and various cellular processes [25,26]. Studies suggest that approximately 60% of PM patients carry a somatic BAP1 mutation, making it an attractive target for potential therapeutic strategies [29]. According to a series of 74 patients evaluated in The Cancer Genome Atlas, 34% of tumors exhibited a combination of the most frequent molecular alterations (BAP1, NF2, and CDKN2A). In a preclinical study, it was observed that when BAP1 alterations were associated with the loss of NF2 and/or CDKN2A, more aggressive tumors developed [100].

The detection of BAP1 mutations presents challenges for clinical application due to the heterogeneity of mutation types and technical limitations for detecting these mutations. Standard sequencing methods such as NGS can miss CNVs or epigenetic silences, which underestimate the presence of the mutation. IHC techniques are a method that is easily replicated in clinical practice, but IHC can miss truncating mutations. If a predictive role of BAP1 for different therapies is confirmed in future clinical practice, it will be necessary to establish recommended guidelines for the detection of BAP1 in PM.

The prognostic significance of BAP1 mutations remains controversial. As reviewed above, all data regarding the prognostic value of BAP1 in patients with PM come from retrospective data, and most included a limited number of patients. Initial studies linked BAP1 loss to improved survival, but subsequent research yielded contradictory results [49,50,51,52,53]. A large meta-analysis involving over 600 patients ultimately determined that BAP1 loss is not directly correlated with survival outcomes [53]. Studies indicate that patients with germline BAP1 mutations tend to be younger, have less aggressive tumors, and respond better to treatment [55,56].

Given that BAP1 is the most recurrent molecular alteration in PM, its predictive value has been explored in the context of approved therapies. Regarding first-line treatment with platinum and pemetrexed, BAP1 loss predicts improved survival [48,56]. In second-line treatments, the results are inconsistent. In particular, studies suggest that BAP1 loss is associated with reduced chemotherapy efficacy when using gemcitabine, whereas, with vinorelbine, the opposite is observed: patients with BAP1 loss show improved survival [60,61,62]. However, these findings are based on a limited number of patients, preventing definitive conclusions.

Immunotherapy is now a standard first-line treatment for PM patients. The predictive value of BAP1 in immunotherapy has been studied, with conflicting results regarding its association with PD-L1 expression [70]. A preclinical study suggests that, regardless of PD-L1 levels, patients with BAP1 loss may exhibit increased sensitivity to immunotherapy [72]. However, these findings have not been clinically confirmed, as randomized first-line trials have yet to report efficacy data for immunotherapy in BAP1-deficient patients. In contrast, in patients with germline BAP1 mutations, immunotherapy appears to be more effective, resulting in improved survival [11].

Several clinical trials have tested therapies that may be effective in BAP1-mutant patients, particularly inhibitors of EZH2 and PARP [64,65,80,85]. Despite promising preclinical evidence, clinical trial results have shown limited activity of these inhibitors in BAP1-mutant patients. Therefore, new research directions are needed, exploring alternative targets such as Hippo pathway inhibitors or novel immunotherapy combinations that could benefit this patient population.

## 10. Conclusions

In conclusion, BAP1 is a frequent molecular alteration in PM but is not a reliable predictive biomarker for treatment response at the moment. BAP1 loss detected by IHC helps to distinguish mesothelioma from other malignancies. Currently, BAP1 determination is recommended by the ASCO and ESMO guidelines to establish the diagnosis of mesothelioma in situ in cases of suspected mesothelioma in patients with pleural effusion [58,59]. However, at the moment, BAP1 loss does not consistently predict response to standard therapy (chemotherapy and immunotherapy) or targeted therapies under development (PARP inhibitors), and, currently, the value of BAP analysis is therefore reserved for research purposes.

Future research is needed to understand the role of BAP1 in the pathogenesis of PM, as well as its potential interactions with other suppressor genes that allow for the selection of targeted therapies. Examining the role of BAP1 in the tumor microenvironment, as well as exploring the influence of BAP1 on inflammatory signaling pathways, could identify patients who are candidates for immunotherapy. The knowledge of the results regarding the sensitivity to immunotherapy of BAP1 patients included in phase III clinical trials could identify patients who are candidates for immunotherapy. Finally, exploring other therapies with activity against DNA repair disorders, such as ATR inhibitors, or even immunotherapy combinations with novel targets, could identify personalized treatment options for patients with BAP1 loss.

## Figures and Tables

**Table 1:** Frequency of cancer types in BAP1 germline mutant families.

Cancer Type	Occurrence of Cancer (%)
Mesothelioma	30
Uveal melanoma	18
Cutaneous melanoma	14
Clear cell renal carcinoma	7
Basal cell carcinoma	6
Breast cancer	6
Lung cancer	3
Meningioma	2
Skin carcinoma	2
Other tumors (thyroid, colon, bladder, gastric, prostate, liver, ovarian, pancreas)	<2

**Table 2:** Prognostic value of somatic BAP1 mutations.

Author	Number p	Prognostic Role	Outcomes
Farzin M (Pathology 2015) [49]	229	+	46% p BAP1 loss BAP1 loss associated with increased survival (16 vs. 6 m)
Forest F (Pathology 2018) [19]	117	+	59% p BAP1 loss BAP1 loss associated with increased survival (18 vs. 12 m)
Murrone A (J Thoracic Disease 2021) [50]	55	+	Only epithelioid tumors 60% p BAP1 loss BAP1 loss associated with increased survival (14 vs. 11 m), not confirmed in multivariate
Pulford E (Markers 2017) [53]	81	-	60% p BAP1 loss BAP1 loss associated with decreased survival (6 vs. 11 m)
Cantini L (Lung Cancer 2020) [54]	60	-	82% p BAP1 loss BAP1 loss associated with decreased survival (5 vs. 18 m)
Cantini L (Lung Cancer 2020) [54]	698	Non-significant	Meta-analysis BAP1 loss associated with non-significant increased survival (18 vs. 12 m)
